# A multi-labeled tree dissimilarity measure for comparing “clonal trees” of tumor progression

**DOI:** 10.1186/s13015-019-0152-9

**Published:** 2019-07-27

**Authors:** Nikolai Karpov, Salem Malikic, Md. Khaledur Rahman, S. Cenk Sahinalp

**Affiliations:** 1 Department of Computer Science, Indiana University, Bloomington, IN USA; 2 School of Computing Science, Simon Fraser University, Burnaby, BC Canada

**Keywords:** Intra-tumor heterogeneity, Tumor evolution, Multi-labeled tree, Tree edit distance, Dynamic programming

## Abstract

We introduce a new dissimilarity measure between a pair of “clonal trees”, each representing the progression and mutational heterogeneity of a tumor sample, constructed by the use of single cell or bulk high throughput sequencing data. In a clonal tree, each vertex represents a specific tumor clone, and is labeled with one or more mutations in a way that each mutation is assigned to the oldest clone that harbors it. Given two clonal trees, our multi-labeled tree dissimilarity (MLTD) measure is defined as the minimum number of mutation/label deletions, (empty) leaf deletions, and vertex (clonal) expansions, applied in any order, to convert each of the two trees to the maximum common tree. We show that the MLTD measure can be computed efficiently in polynomial time and it captures the similarity between trees of different clonal granularity well.

## Introduction

According to the *clonal theory of cancer evolution* [1], cancer originates from a single cell which had acquired a set of mutations that provide it proliferative advantage compared to the neighboring healthy cells. As tumor grows, cancer cells acquire new mutations and some of them might accumulate a set of mutations conferring further selective advantage or disadvantage compared to the other cells. This continues over a period of time and at the time of the clinical diagnosis, tumors are usually heterogeneous consisting of multiple cellular populations, harboring distinct sets of mutations, leading to different phenotypes. Each such cellular population is considered to be a clone.

**Fig. 1:**
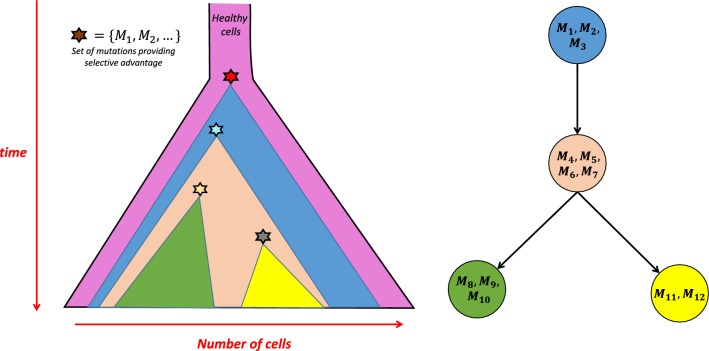
Graphical overview of tumor initiation and growth (left) and the corresponding clonal tree of tumor evolution (right). Sets of mutations providing proliferative advantage and driving the emergence of new clones are denoted as stars in the left and as sets of corresponding mutations in the right panel (e.g. red star from the left panel represents the set of mutations $\{M_{1}, M_{2}, M_{3}\}$.) Vertex corresponding to the healthy cells is omitted as it would be non-informative

The whole process of tumor initiation and growth is illustrated in Fig. 1 (left panel).

One of the most widely used ways of depicting mutational heterogeneity and tumor progression over time is by the use of a *clonal tree of tumor evolution*. Here, each individual vertex represents a distinct clone and each mutation (i.e. its label) is placed as part of the label of clone where it occurs for the first time in evolutionary history. In this work we focus on trees built by the use of single nucleotide variants (SNVs), which represent the most widely used type of mutations in reconstructing trees of tumor evolution [2]. We also assume that each SNV occurs exactly once during the course of tumor evolution and never gets lost (infinite sites assumption, usually abbreviated as ISA). Some recently introduced methods (e.g. SiFit [3]) allow for the violations of ISA and, in such cases, we expect that labels corresponding to mutations violating ISA are removed from the trees prior to dissimilarity calculation. In order to simplify our figures, in each figure in this work we omit the vertex representing population of healthy cells. Namely, such vertex would be non-informative as it would always be label-free (since healthy cells are assumed to contain none of the mutations relevant to cancer progression) and attached as the parent of root vertex in each of the figures presented in this work. See Fig. 1 for an illustration of tumor growth (left panel) and the corresponding clonal tree of tumor evolution (right panel). Note that the children of a vertex in a clonal tree are unordered.

A popular alternative to the clonal tree is the *mutation tree*, a special case of the clonal tree, where the label of each vertex consists of exactly one mutation [4, 5]—thus a mutation tree is a clonal tree with the highest possible granularity. As can be expected, any clonal tree can be easily converted to the mutation tree as follows. Consider an arbitrary edge (*u*, *v*) and assume without loss of generality (WLOG) that a set of all mutations assigned to it is $\left\{ M_{1}, M_{2}, \dots , M_{k}\right\}$. Now replace edge (*u*, *v*) by a path with vertices $\{w_{0}=u, w_{1}, w_{2}, \dots , w_{k-1}, w_{k}=v\}$ and edges $\{(w_{0},w_{1}), (w_{1}, w_{2}), \dots , (w_{k-1}, w_{k})\}$, such that exactly one mutation, WLOG $M_{i}$, is assigned to the edge $(w_{i-1}, w_{i})$ for each $i \in \{1,2, \dots , k\}$. Note that from a given clonal tree which is not mutation tree (i.e. contains at least one vertex with two or more labels), multiple different mutation trees can be obtained. More precisely, from the above, it is obvious that any vertex with *k* mutations assigned to it can be expanded to a chain of *k* vertices, each having exactly one mutation as its label, in *k*! different ways. Consequently, considering the numbers of mutations assigned to vertices of the clonal tree *T* and taking the product of factorials of these numbers gives a formula for computing the exact number of different mutation trees that can be obtained from *T*.

There are additional tree representations [5] for tumor evolution but in this work we focus on clonal trees only.

### (Dis)similarity measures between tree representations of tumor evolution

In the past few years, we have witnessed rapid developments in computational methods for inferring trees of tumor evolution from both bulk and single cell high throughput sequencing (HTS) data [4–15].

In order to assess the accuracy of the proposed method, many of these studies use simulated HTS data extracted from synthetic tumor compositions. The inferred tree is then compared against the (synthetic) ground truth. We will call the *ground truth tree* the *true tree*. Other studies, such as the Pan Cancer Analysis of Whole Genomes Project (PCAWG) compare trees inferred by participating methods on real tumor samples to reach a consensus tree. In order to compare clonal trees with varying granularity (granularity can be measured in terms of the average number of mutations assigned to a clone) the measure(s) used should be versatile enough to discriminate real topological differences between trees from those differences due to the type and coverage of the HTS data used by a method; e.g. such a “dissimilarity” measure should be equal to 0 between any clonal tree and its corresponding mutation tree (obtained using the procedure described above).

Unfortunately, comparing trees of tumor evolution is a challenging problem and available measures fail to fully capture (dis)similarities between inferred and true trees. Many of existing measures only aim to compare the relative placement of pairs of mutations across two trees, e.g. whether the two mutations maintain an ancestor–descendant relationship in both trees (we discuss several of the existing measures in more detail in "The existing measures and their limitations" section). Such measures can not capture topological differences between distinct trees, e.g. a simple topology with two vertices, where all but one of the mutations is assigned to the non-root vertex, v.s. a star topology where each vertex is assigned a single mutation. Thus measures of tree similarity that not only consider the relative placement of mutations but also the topological structure of the trees are of high demand.

The standard measure to compare combinatorial objects—such as strings, especially in bioinformatics, is the edit distance. This measure has numerous applications and a large number of variants, not only for strings but also for labeled trees, have been considered in the past. The classical Levenstein edit distance between two strings is defined as the minimum number of single symbol deletions on the two strings so that what remain from the strings are identical (in fact the longest common subsequence of the two strings). As such, it has a well established dynamic programming algorithm (e.g. [16]). The running time of this algorithm is proportional to the product of the lengths of the two input strings and the existence of a sub-quadratic algorithm is unlikely [17]. In general, the complexity of computing an edit distance strictly depends on the set of allowed edit operations. E.g. if we consider a variant of the problem where only single character mismatches and block reversals are allowed, then the running time reduces to $O(n\log ^2{n})$ [18]—here *n* is the total length of the strings; on the other hand, the variant where only mismatches, block deletion and move operations are allowed is *NP*-hard  [19].

Extensions of edit distance measures for rooted trees have typically been defined for trees with ordered vertices, each with a single label, where the goal is to transform one tree to the other by the use of vertex deletions (or, equivalently, vertex insertions) and vertex label replacements [20]. Based on such tree edit distance measures, a notion of tree alignment has also been introduced, both for vertex ordered as well as unordered trees [21]. For many of the vertex ordered cases, there are polynomial time algorithms that can solve the distance/alignment problem [20–29], whereas for several unordered cases, the both the alignment and the corresponding tree edit distance problems are NP-hard [30, 31] or MAX SNP-hard [21, 32].

Motivated by the Levenshtein edit distance between strings, edit distances for trees with unordered vertices are defined in relation to the *largest common subtree* [32] between the input trees: here the goal is to perform the minimum number of label deletions (and eliminate the resulting empty nodes) from the two input trees so that the remaining subtrees are identical. The notion of the largest common subtree of two trees and the implied edit distance can be generalized to clonal (multi-label) trees. Unfortunately, just like other edit distances for unordered trees [33], this distance would be NP-hard (in fact MAX SNP-hard) to compute. Moreover, none of the results in the literature deal with trees where vertices may have more than a single (mutational) label—as is the case for the clonal tree comparison problem.

In this paper we consider a restricted version of the above notion of tree edit distance by allowing label (and implied node) deletions for leaves. This notion of distance can naturally be generalized to multi-labeled trees and the resulting “dissimilarity” measure (multi-labeled tree dissimilarity, MLTD) can be computed in polynomial time. More importantly, it successfully captures the differences between clonal trees: for example it satisfies a key condition that two clonal trees from which it is possible to produce two identical mutation trees have a dissimilarity of 0.

Multi-labeled tree dissimilarity is the first polynomial time computable dissimilarity measure for vertex unordered trees.^1^ We have devised and implemented an algorithm to compute MLTD and applied it to a number of synthetic and real data sets to compare trees inferred by some of the available tumor history reconstruction methods with success.

## Definitions

While this work is motivated and currently has the main application in the comparison of clonal trees of tumor evolution, possible novel applications may arise in the future. In order to minimize the background knowledge of cancer evolution and related terminology required to follow description of the presented algorithms, in this section we first provide formal definition of *multi-labeled tree* and use this term throughout the sections containing algorithms description ("Definitions", "Set alignment problem" and  "Computing a maximum common tree in 2 the general case" sections). Second, we describe how the dissimilarity measure between two arbitrary multi-labeled trees is computed. Finally, for the readers interested in the presented practical application, we also provide motivation for the introduced multi-labeled tree and edit operations.

### Multi-labeled tree

A rooted tree $T=(V,E)$ is a connected, acyclic, undirected graph with set of vertices *V* (also denoted as *V*(*T*)) and edges *E* (also denoted as *E*(*T*)), with a particular vertex *r* identified as the root. For each non-root vertex *v*, any vertex *u* that lies on the simple path between *v* and the root is considered to be its ancestor; in particular, the vertex $u=p(v)$ on this path which has an edge to *v* is considered to be its parent. The depth of vertex *v* denoted *d*(*v*), is thus defined as the number of its ancestors. The lowest common ancestor of any pair of vertices *u* and *v*, denoted ${\text {lca}}(u, v)$, is defined as a common ancestor of both *u* and *v* whose depth is maximum possible. The structure of a tree induces partial order $\preceq$ on its vertices: $u \preceq v$ denotes that *u* is an ancestor of *v*.

*Multi-labeled tree* *T* is a rooted tree in which each vertex *v* other than root has a subset $L_v$ of labels from a universe $\mathbb {L}$ and each label is unique to a vertex, i.e. $L_{u} \cap L_{v} = \emptyset$ for each pair of distinct vertices *u* and *v*. We denote the set of all labels assigned to the vertices of *T* as *L*(*T*). In other words, $L(T) = \bigcup \nolimits _{v \in V(T)} L_{v}$.

### MLTD measure between two multi-labeled trees

Consider the following types of edit operations on multi-labeled tree:

- *deleting a label* where one of the labels is removed from some set $L_v$,
- *deleting an unlabeled leaf* where a vertex is removed from the tree. This operation is allowed to be performed only for unlabeled leaves, i.e. vertices with no labels and no children,
- *expanding a vertex* where vertex *v* is replaced by two vertices $v_1$ and $v_2$ such that all children of *v* after this operation are children of $v_2$, and the parent of *v* is the parent of $v_1$, and $v_1$ is the parent of $v_2$. Each of the labels from $L_{v}$ is assigned to exactly one of the $L_{v_{1}}$ and $L_{v_{2}}$.

A *Common tree* of arbitrary multi-labeled trees $T_{1}$ and $T_{2}$ is any multi-labeled tree which can be obtained from each of $T_{1}$ and $T_{2}$ by the use of edit operations defined above. A *maximum common tree* of $T_{1}$ and $T_{2}$ is a common tree of $T_{1}$ and $T_{2}$ having the largest number of labels among all common trees of $T_{1}$ and $T_{2}$. We define MLTD measure between $T_{1}$ and $T_{2}$ as the difference between the total number of labels in $T_{1}$ and $T_{2}$ and twice the number of labels in their maximum common tree. In other words, MLTD is defined as the total number of labels required to be removed from the two trees in the process of obtaining their maximum common tree.^2^ For two trees given as an input, finding their maximum common tree obviously suffices to compute MLTD and will therefore be the main focus of our algorithms described below.

As mentioned earlier MLTD defined above is not a metric since it is akin to the “inverse set intersection” and thus does not satisfy the triangle inequality. For example, given $\mathbb {L}=\left\{ A,B\right\}$ and the following trees: (i) tree $T_{1}$ consisting of two vertices, labeled by *A* (root vertex) and *B* (non-root vertex) (ii) tree $T_{2}$ consisting of two vertices, labeled by *B* (root vertex) and *A* (non-root vertex) and (iii) a single vertex tree $T_{3}$ where vertex label consists of both, *A* and *B*, MLTD between $T_{1}$ and $T_{3}$, as well as $T_{2}$ and $T_{3}$, equals 0, whereas MLTD between $T_{1}$ and $T_{2}$ equals 2.

### (Dis)similarity between multi-labeled trees in the context of tumor evolution

Formal definition of multi-labeled tree presented above is motivated by the clonal tree of tumor evolution discussed in "Introduction" section. In a clonal tree, root vertex *r* represents population of healthy cells and each non-root vertex represents tumor clone. Universe $\mathbb {L}$ represents set of mutations detected in a given tumor and $L_{v}$ denotes the set of mutations appearing for the first time at vertex (clone) *v*. The constraint $L_{u}\cap L_{v}=\emptyset$ for each pair of distinct vertices *u* and *v*, ensures that each mutation appears at most once during the course of tumor evolution (this follows directly from the ISA).

The main difference between multi-labeled and clonal tree is that in the latter we have constraint that the set of labels assigned to the root vertex *r* is empty (since this vertex represents population of healthy cells which is assumed to be mutation-free) and $L_{v}\ne \emptyset$ for each $v\in V(T)\backslash \{ r\}$. Namely, if *v* is non-root vertex such that $L_{v}$ is empty then clone *v* would be, with respect to the set of mutations it harbors, identical to its parent which is atypical for clonal trees as it introduces unnecessary redundancy in representation of the process of tumor evolution. For the simplicity, in the figures of clonal trees presented in this work we do not show a root since its set of labels is empty hence such vertex would be non-informative.

Note that any multi-labeled tree can be converted to a unique clonal tree using the following steps: (i) merging each of non-root vertices having empty set of labels with its parent and repeating this until each non-root vertex has non-empty set of labels and (ii) in the case that root of the tree obtained after the first step has non-empty set of labels, add a new vertex without any mutational labels and connect it to the root of the modified tree (so that it becomes new root). In the applications, we first consider each clonal tree as a mutation tree and, once a common tree is obtained, it is converted to a clonal tree using the two of these steps.

While the notion for the edit operation of label deletion is intuitively clear as in general case one would be unable to obtain a common tree without allowing this or any similar operation which removes some labels, the edit operation of expanding a vertex at no cost is directly motivated by the existing different ways of representing clonal trees of tumor evolution. More precisely, we introduce it in order to be able to capture differences between two clonal trees which are due to different levels of granularity in tree representation. An example of such trees is shown in Fig. 6 where tree of tumor evolution is shown in (a) and its more refined versions are shown in (c) and (d) (more detailed discussion of Fig. 6 is provided in "The existing measures and their limitations" section). Finally, the operation of deleting an unlabelled leaf is introduced in order to allow obtaining common tree of trees having certain topological differences, mostly in terms of branching. For example, if we are given a linear and non-linear clonal tree as two input trees, they can not be reduced to a common tree using solely the label deletion and vertex expansion operations. Also, note that deletion of unlabeled leaf requires deletion of all of its labels prior to applying this edit operation which is usually costly. However, this is desired when computing a “dissimilarity” between clonal trees of tumor evolution since the placement of mutations on vertices from different branches (i.e. to the clones from different lineages) in one clonal tree and to the vertices that in the ancestor–descendant relation (i.e. to the clones from the same lineage) in the second clonal tree represents fundamental dissimilarity between the two trees and needs to have an appropriate contribution to their “dissimilarity”.

## Set alignment problem

We first demonstrate how maximum common tree is computed for a pair of trees where each tree is a path. Obviously in this case any common tree between the input trees is also a path. Let the ordered sequence of vertices of the first tree/path be $v_{1},v_{2}, \dots , v_{n}$ with respective label sets $S_{1}, S_{2}, \dots , S_{n}$, and the ordered sequence of vertices of the second tree/path be $w_{1}, w_{2}, \dots , w_{m}$ with respective label sets $P_{1}, P_{2}, \dots , P_{m}$. (Assume that $S_i, P_j$ are subsets of $\mathbb {L}$ and that any label $u \in \mathbb {L}$ occurs exactly in one of $S_{1},S_{2}, \ldots , S_{n}$ and exactly in one of $P_{1}, P_{2}, \ldots , P_{m}$.) Let $f :\mathbb {L} \rightarrow \{1,2,\ldots , n\}$ and $g : \mathbb {L} \rightarrow \{1, 2, \ldots , m\}$ be the functions that map labels to vertex indices, respectively in the first and the second tree such that $v_{f(a)}$ denotes the vertex of label *a* in the first tree and $w_{g(a)}$ denotes the vertex of the label *a* in the second tree.

It is easy to see that computing a maximum common tree in this special case is equivalent to the following generalized version of the string edit distance problem for a pair of ordered sets.

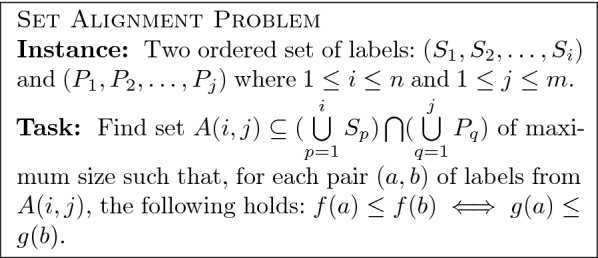



The following lemma offers an efficient algorithm for solving the Set Alignment Problem. Our approach for computing dissimilarity between two arbitrary trees (presented in "Computing a maximum common tree in the general case" section) uses this algorithm as a subroutine.

### **Lemma 1**

*Let* ${\text {D}}(i,j)$ *be the size of the set which is answer of the* Set Alignment Problem *for the instance where input sequences are* $(S_1, \ldots , S_i)$ *and* $(P_1, \ldots , P_j)$ (*i.e. according to the notation from the above* $D(i,j) = \left| A(i,j)\right|$). *Then the following hold:*

- ${\text {D}}(i, 0) = {\text {D}}(0, j) = 0$, *for all non-negative integers* *i* *and* *j*.
- ${\text {D}}(i, j) = \max \left( {\text {D}}(i, j - 1), {\text {D}}(i - 1, j)\right) + |S_i \cap P_j|$, *for all positive integers* *i* *and* *j*.

### *Proof*

The first equation easily follows from the fact that $A(i,0) \subseteq \emptyset$ and $A(0,j) \subseteq \emptyset$.

For the second equation, we first prove that ${\text {D}}(i, j) \ge \max ({\text {D}}(i, j - 1), {\text {D}}(i - 1, j)) + |S_i \cap P_j|$. In order to prove this, observe that each of $A(i,j-1) \cup (S_i \cap P_j)$ and $A(i-1,j) \cup (S_i \cap P_j)$ represent a valid candidate solution for the instance of Set Alignment Problem with the input sequences $(S_1, \ldots , S_i)$ and $(P_1, \ldots , P_j)$. Namely, in the case of set $A(i,j-1) \cup (S_i \cap P_j)$ (analogous applies to the set $A(i-1,j) \cup (S_i \cap P_j)$), if we consider two arbitrary labels *a* and *b* of this set, then:

- If $a \in A(i,j-1)$ and $b \in A(i,j-1)$ then $f(a) \le f(b) \iff g(a) \le g(b)$ holds by the definition of $A(i,j-1)$.
- If $a \in A(i,j-1)$ and $b \in S_i \cap P_j$ then $f(a) \le i$ and $g(a) \le j-1$. On the other hand, $f(b)=i$ and $g(b)=j$ hence $f(a) \le f(b) \iff g(a) \le g(b)$ is obviously satisfied.
- Case where $a \in S_i \cap P_j$ and $b\in A(i,j-1)$ is analogous to the previous case.
- Case where both *a* and *b* are from $S_i \cap P_j$ is trivial since in this case $f(a)=f(b)=i$ and $g(a)=g(b)=j$ implying that $f(a) \le f(b) \iff g(a) \le g(b)$ holds in this case as well.

Now it suffices to prove that ${\text {D}}(i, j) \le \max ({\text {D}}(i, j - 1), {\text {D}}(i - 1, j)) + |S_i \cap P_j|$. In order to prove this, consider the partition of *A*(*i*, *j*) into $A(i,j) \backslash (S_i \cap P_j)$ and $S_i \cap P_j$. We claim that at most one of the sets $S_i$ and $P_j$ has non-empty intersection with the set $A(i,j) \backslash (S_i \cap P_j)$. To prove this, assume on contrary that there exists $a \in S_i \cap \left( A(i,j) \backslash (S_i \cap P_j)\right)$ and $b \in P_j \cap \left( A(i,j) \backslash (S_i \cap P_j)\right)$. Since $a \in S_i$ we have $f(a)=i$. For *b* we have that $b \in A(i,j)$ and $b \notin S_{i}$ implying that $f(b) \le i-1$. Similarly, $g(a)\le j-1$ and $g(b)=j$. By the above assumption, both *a* and *b* belong to *A*(*i*, *j*) but obviously they violate constraint $f(a)\le f(b) \iff g(a) \le g(b)$ which is, by definition of *A*(*i*, *j*) satisfied for all of its labels. This contradiction directly implies our latest claim. To finalize the proof of inequality ${\text {D}}(i, j) \le \max ({\text {D}}(i, j - 1), {\text {D}}(i - 1, j)) + |S_i \cap P_j|$ assume WLOG that the intersection of $S_i$ and $A(i,j) \backslash (S_i \cap P_j)$ is the empty set. This implies that *A*(*i*, *j*) does not contain any label from $S_{i} \backslash (S_{i} \cap P_{j})$. Therefore $D(i,j) \le D(i-1,j) + \left| S_{i} \cap P_{j}\right| \le \max ({\text {D}}(i, j - 1), {\text {D}}(i - 1, j)) + |S_i \cap P_j|$ which completes our proof. $\square$

Lemma 1 provides a dynamic programming formulation for calculating “dissimilarity” *D*(*n*, *m*) between trees $T_1$ and $T_2$.

### **Observation 1**

*Total time and total space required for calculating number of labels in each of the sets* $S_i \cap P_j$, *where* $i \in [n]$ *and* $j \in [m]$ *are both* $O(\sum \nolimits _{i=1}^n|S_i| + \sum \nolimits _{j=1}^{m}|P_j| + nm)$.

### *Proof*

For each label from $u \in L$ we can store two indices *f*(*u*) and *g*(*u*). This can be implemented in the above time and space by using a hash table. If we know these indices, we can fill the table $I_{ij}$, where $I_{ij} = |S_i \cap P_j|$, by iterating through elements of $\mathbb {L}$ and increasing the value of $I_{f(x)g(x)}$ by one for each $x \in \mathbb {L}$. $\square$

### **Lemma 2**

*The* Set Alignment Problem  *is solvable in* $O \left(\sum \nolimits _{i=1}^n|S_i| + \sum \nolimits _{j=1}^{m}|P_j| + nm \right)$ *time and space.*

### *Proof*

Follows straightforwardly from Lemma 1 and Observation 1. $\square$

## Computing a maximum common tree in the general case

We now describe an efficient algorithm for computing a maximum common tree. Note that in the remainder of the paper we call all vertices in a tree with exactly one child as *non-crucial* vertices and all other vertices, i.e. leaves, and vertices with two or more children, as *crucial* vertices. Now consider the sequence of edit operations applied to a tree $T_1$ in the process to reaching a common tree *T* with another tree $T_2$.

### **Observation 2**

*Each edit operation applied to any vertex creates at most one (new) crucial vertex; no edit operation can increase the total number of crucial vertices.*

### *Proof*

The proof is based on analyzing the effect that application of a given edit operation might have on the set of crucial vertices.

**Fig. 2:**
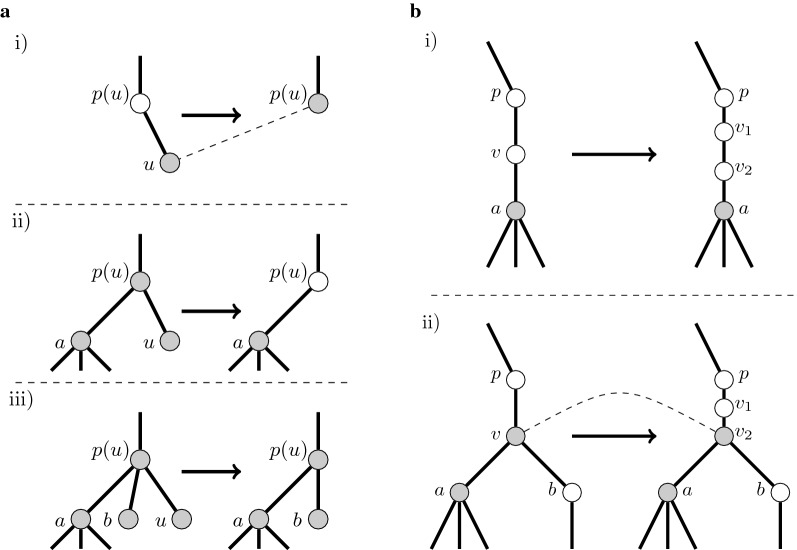
**a** It shows how the set crucial vertices from Observation 2 changes after deleting a leaf *u*. We use dashed lines to denote correspondence among *u* and a vertex in a tree obtained by an edit operation. Only in the case (i), when a *u* was an unique child of *p*(*u*), *u* corresponds to *p*(*u*) in a tree after deletion of *u*. In other cases *u* does not correspond to any vertex in a new tree. In the case (ii) a vertex *p*(*u*) lost the status of a crucial in a tree after deletion and also does not correspond to the copy of himself in a new tree. In the case (iii) the vertex *p*(*u*) keep the status of a crucial and vertex and corresponds to the copy of himself. It is easy to see that the status of other vertices still unchanged and all vertices except *p*(*u*) corresponds to copies of himself in a new tree.** b** The figure illustrate changing a tree after expanding a vertex *v* into $v_1$ and $v_2$. We use dashed lines to denote correspondence between *u* in a tree before operation and a vertex in a new tree. In the case (i) *v* is non-crucial and both copies of *v* stays non-crucial. In the case (ii) a crucial vertex *v* corresponds to a crucial vertex $v_2$
- Finally, the edit operation of expanding, i.e., splitting a vertex *v* into $v_1$ and $v_2$ does change the topology of the tree (i) but it does not create a new crucial vertex if *v* is non-crucial; however, (ii) if a vertex *v* is crucial, then $v_2$ becomes crucial after the edit operation, but $v_1$ stays non-crucial. See Fig. 2b for examples.

- The edit operation of deleting a label does not change the topology of the tree or the set of crucial vertices in the tree.
- The edit operation of deleting a leaf *u* does change the topology of a tree, but with respect to the set of crucial vertices, the only update is that *u* is lost, and, (i) provided that *u* was the only child of *p*(*u*), *p*(*u*) becomes crucial, or (ii) provided that *u* was one of the two children of *p*(*u*), *p*(*u*) becomes non-crucial, or (iii) provided that *u* was one of more than two children of *p*(*u*), *p*(*u*) stays crucial. All other vertices remain unaltered. See Fig. 2a for detailed examples.

In summary, after an arbitrary edit operation, at most one new vertex is added to the set of crucial vertices. However, in the case that new crucial vertex is added, at least one of such vertices is deleted implying that the total number of crucial vertices never increases. $\square$

The observation above indicates that an edit operation applied to a crucial vertex *u* may create a new crucial vertex *v*. In that case, we say that the crucial vertex *u* in $T_1$ *corresponds to* a crucial vertex *v* in $T_1'$ (if latter was created). In case of an expansion of vertex *u* in $T_1$ to two vertices $u_1$ and $u_2$, we say that *u* corresponds to $u_2$ in $T_1'$. In case of a deletion of a leaf *u*, if *p*(*u*) which was originally non-crucial, became crucial, then we say that *u* in $T_1$ corresponds to *p*(*u*) in $T_1'$. For any vertex *v* which remains unedited and crucial in $T_1'$, we say that *v* in tree $T_1$ corresponds to *v* in the tree $T_1'$.

Finally, we say that *v* in $T_1$ corresponds to $v'$ in *T* if for the sequence of trees $T_1=T^0_1, T^1_1, \ldots , T^l_1=T$ (where $T^{i+1}_1$ is obtained from $T^i_1$ by an edit operation) there exists the sequence of vertices $v=v^0, v^1, \ldots , v^l=v$ (where $v^l \in V(T^l_1)$) such that $v^{i}$ corresponds to $v^{i+1}$ for all *i*. We extend the notion of correspondence to $T_2$ in a similar manner.

Thus we notice the following fact.

### **Observation 3**

*We can construct the correspondence between a subset of crucial vertices in* $T_1$ *and* $T_2$ *and crucial vertices in the common tree. Such that each crucial vertex in the common tree corresponds to some vertex in* $T_1$ *and* $T_2$.

Given trees $T_1$ and $T_2$, their common tree *T* and the vertices in $T_1$ and $T_2$ that correspond to every crucial vertex in *T*, it is straightforward to establish the edit operations to transform $T_1$ and $T_2$ to *T*. The algorithm to compute *T* makes use of this observation.

### **Observation 4**

*Given two sets of crucial vertices* $u_1, \ldots , u_l$ *and* $v_1, \ldots , v_l$ *in* $T_1$ *and* $T_2$ *respectively such that* $u_i$ *and* $v_i$ *correspond to same crucial vertex in the common tree* *T* *for each* *i*, *we can reconstruct a common tree* $T'$ *such that the number of labels in* $T'$ *is at least that in* *T*.

### *Proof*

**Fig. 3:**
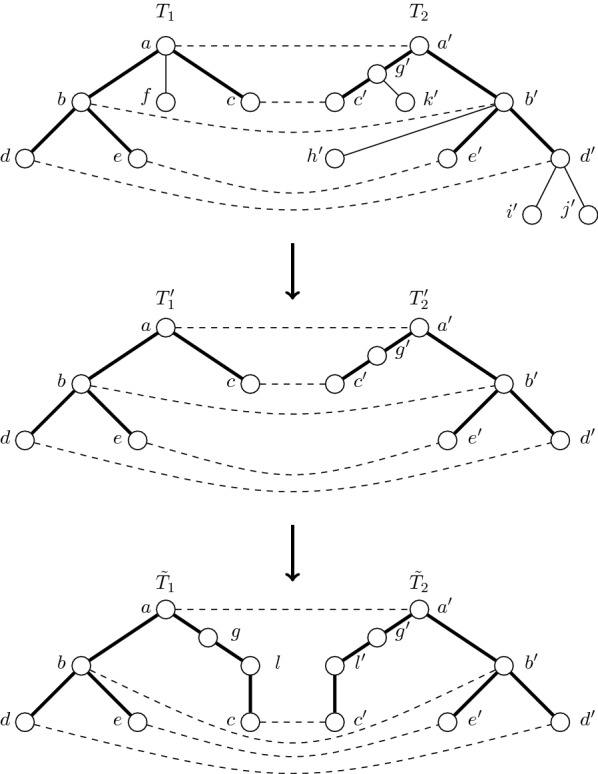
Illustrates how to obtain a maximum common tree of trees $T_1$ and $T_2$. We used dashed lines to denote pairs of vertices $u_i, v_i$ from the proof of Observation 4. After the first step of proof we delete all vertices which do not belong to paths from roots to some crucial vertex and obtain from trees $T_1$ and $T_2$ trees $T'_1$ and $T'_2$ which are topologically isomorphic to each other. After applying the step two from proof we obtain by applying sequence of optimal operations to pairs of paths $((a), (a')), ((c, g), (c', g')), ((b), (b')), ((d), (d')), ((e), (e'))$ from $T'_1$ and $T'_2$ trees $\tilde{T}_1$ and $\tilde{T}_2$ which are equal to each other and contain a maximum number of labels

**Fig. 4:**
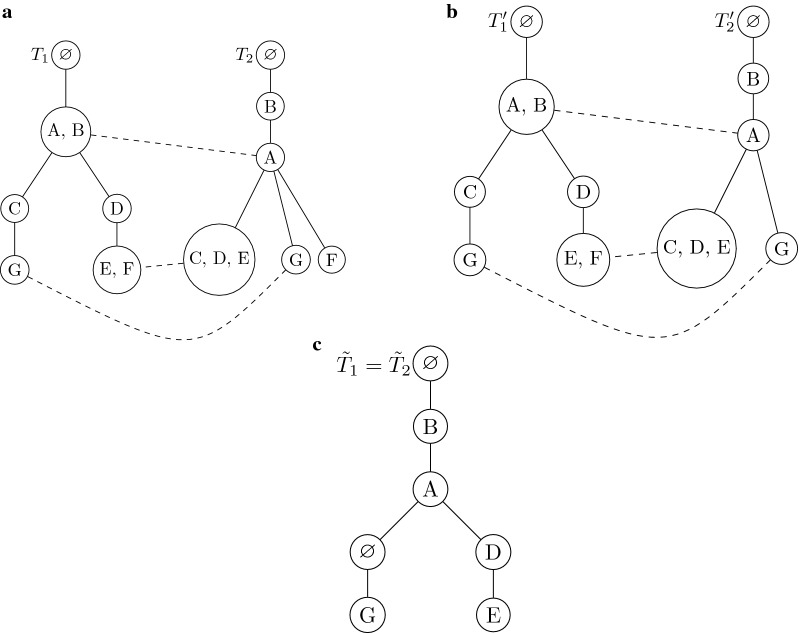
**a** $T_1$ and $T_2$ before applying the first step from Observation 4,** b** $T'_1$ and $T'_2$ obtained from $T_1$ and $T_2$ from ** a** after first step of deleting vertices which do not belong to paths between root and crucial vertices,** c** the resulting tree $\tilde{T}_1 = \tilde{T}_2$ after applying second step

Here we describe the procedure of reconstructing the tree $T'$ in two steps (see Figs. 3 and 4 as illustrations).

In the first step we delete each label which cannot belong to *T* in a trivial manner: let $S_1$ ($S_2$) be the set of vertices which do not lie on a path from the root of $T_1$ ($T_2$) to some $u_i$ ($v_i$). Then we delete all vertices from $S_1$ (and $S_2$) together with their labels. Note that no label which is present in tree *T* will be deleted: if a vertex *v* does not belong to a path from the root to some crucial vertex in *T*, then any label from $L_v$ cannot be present in *T*. However, if any label in *T* that is in $L_v$ for some vertex *v* which lies on a path from the root to a leaf *w* (which is necessarily crucial) then there must exist a pair of vertices $u_i, v_i$ which correspond to the leaf *w*.

Thus, starting from the leaf level, we can delete all vertices which do not belong to a path from the root to any $u_i$ (and $v_i$). It is easy to see that this first step transforms $T_1$ and $T_2$ into isomorphic trees. Let $r_i$ denote the root of tree $T_i$; the isomorphism $\phi$ on $r_1, u_1, \ldots , u_l$ which transforms $T_1$ into $T_2$ is $\phi (r_1)=r_2, \phi (u_1) = v_1, \ldots , \phi (u_l) = v_l$.

Let $T_1'$ and $T_2'$ denote the trees respectively produced from $T_1$ and $T_2$ after applying the first step. Notice that, $T_1'$ and $T_2'$ are also topologically isomorphic to *T* and $T'$.

In the second step, for each pair of vertices $v_i$ and $u_i$ we consider the pair of “maximum” paths from $v_i$ and $u_i$ to the associated root, which do not contain other vertices from $v_1, \ldots , v_l$ and $u_1, \ldots , u_l$. For this pair of paths we apply a sequence of edit operations that expand vertices and delete labels, such that the resulting paths will be identical with the maximum possible number of labels.

$T'$ is the tree produced as a result of the second step. Note that on any pair of paths from the vertex pair $u_i$ and $v_i$ to the respective root, the set of labels observed will be identical. This implies that $T'$ is a common tree with number of labels necessarily lower bounded by that of *T*. $\square$

The above observation implies that we can reduce the problem of computing a maximum common tree between two multi-labeled trees to the problem of finding an *optimal pair of sequences* of vertices $u_1, \ldots , u_l$ and $v_1, \ldots , v_l$ corresponding to the maximum common tree.

Our general algorithm for computing the “dissimilarity” between two multi-labeled trees requires constant time access to the solutions to many instances of the Set Alignment Problem, which we compute in a preprocessing step.

Solving Set Alignment Problem  for all pairs of sequences $u_1, \ldots , u_l$ and $v_1, \ldots , v_l$ is impractical. Fortunately, special conditions with respect to the structure of these sequences help us develop an efficient algorithm for finding an optimal pair of sequences as explained below.

The algorithm for computing an optimal pair of sequences will need the solutions to Set Alignment Problem  for all possible downward paths; we call this auxiliary problem Pairwise Alignments on a Tree.

Given a pair of vertices *u*, *v* such that $u \preceq v$, let the following sequence of sets of vertex labels be denoted as ${\text {P}}(u, v) = (L_{w_1}, \ldots , L_{w_k})$ where $w_1 (=u), w_2, \ldots , w_k(=v)$ is called the downward path between *u* and *v*. Then we can define Pairwise Alignments on a Tree  problem formally as follows.

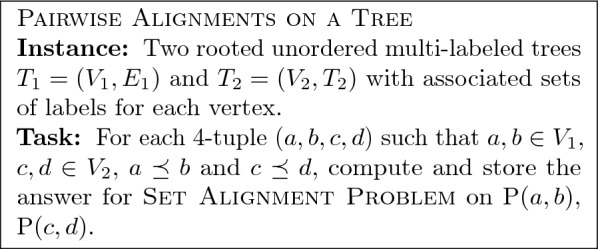



In the next lemma, we introduce equations for computing Pairwise Alignments on a Tree  which forms the basis of our dynamic programming algorithm.

### **Lemma 3**

*Given* $a,b \in V(T_1)$; $c, d \in V(T_2)$; $a \preceq b$; $c \preceq d$, *let* ${\text {D}}(a,c,b,d)$ *be the solution for the instance* ${\text {P}}(a,b)$, ${\text {P}}(c,d)$ *of* Set Alignment Problem. *Then*

1. *If* $a = b$ *and* $c = d$ *then* ${\text {D}}(a,c,b,d)=|L_b \cap L_d|$.
2. *If* $a = b$ *and* $c \ne d$ *then* ${\text {D}}(a,c,b,d) = {\text {D}}(a,c,b,p(d)) + |L_b \cap L_d|$.
3. *If* $a \ne b$ *and* $c = d$ *then* ${\text {D}}(a,c,b,d) = {\text {D}}(a,c,p(b),d) + |L_b \cap L_d|$.
4. *Otherwise* $${\text {D}}(a,c,b,d) = \max ({\text {D}}(a,c,p(b),d),{\text {D}}(a,c,b,p(d))) + |L_b \cap L_d|$$.

### *Proof*

Each of the cases above holds true as a direct consequence of Lemma 1. $\square$

Through a straightforward application of the above lemma, we obtain the following.

### **Lemma 4**

*If* $I_1$ *and* $I_{2}$ *denote the heights of* $T_{1}$ *and* $T_{2}$, *respectively,* Pairwise Alignments on a Tree *is solvable in* $O\left( |V_1||V_2|I_1I_2 + |L(T_1)| + |L(T_2)| \right)$ *time and space.*

### *Proof*

The algorithm is a straightforward implementation of Observation 1 and Lemma 3. Namely, from Observation 1 it follows that the values of $|L_a \cap L_b|$, for all $a \in V_1$ and $b \in V_2$, can be computed by the use of algorithm having time and space complexity $O\left( |V_1||V_2| + |L(T_1)| + |L(T_2)| \right)$. After computing these values, all entries in ${\text {D}}$ can be computed in the time and space that are proportional to the number of all possible combinations of *a*, *b*, *c*, *d*, which is bounded by $|V_1||V_2|I_1 I_2$. Now, combining the above with the obvious inequality $|V_1||V_2|I_1 I_2 \ge |V_1||V_2|$, we have that the overall time and space complexity of the proposed algorithm is $O\left( |V_1||V_2|I_1I_2 + |L(T_1)| + |L(T_2)| \right)$. $\square$

Given a common tree *T* for $T_1$ and $T_2$, let $M : V(T_1) \cup V(T_2) \rightarrow V(T_1) \cup V(T_2)$ be the (partial) bijective mapping between those vertices *v* in $T_1$ and *w* in $T_2$, which correspond to crucial vertices in *T*, such that $M(v)=w$ and $M(w)=v$ only if *v* and *w* have the same crucial vertex in *T*.

### **Observation 5**

*For any pair of vertices* $a, b \in V_1$ (*or* $V_2$) *which correspond to a vertex in the common tree the lowest common ancestor of* *a* *and* *b*, *namely* ${\text {lca}}(a,b)$, *has a mapping*, $M({\text {lca}}(a, b))$ *which is equal to* ${\text {lca}}(M(a), M(b))$. *For any triplet of vertices* $a, b, c \in V_1$ (*or* $V_2$), *the lowest common ancestor of* *a*, *b* *is equal to the lowest common ancestor of* *b*, *c* *if and only if* ${\text {lca}}(M(a),M(b))={\text {lca}}(M(b), M(c))$.

### *Proof*

The observation follows straightforwardly from the construction of correspondence. For that notice that the least common ancestor of vertices can correspond only the least common ancestor in the common tree because we may apply only operations of expanding for internal vertices. $\square$

We now present our algorithm for computing the size of a maximum common tree, which is a combination of dynamic programming and an algorithm for finding a maximum cost matching.

### **Theorem 1**

*The mapping which corresponds to a maximum common tree can be computed in time* $O(|V_1||V_2|(|V_1|+|V_2|)\log (|V_1|+|V_2|) + |V_1||V_2|I_1I_2 + |L(T_1)| + |L(T_{2}|)$.

### Proof

For $i \in \{1,2\}$ and $x \in V_{i}$, let $T_{i}(x)$ be the subtree of $T_i$ rooted at vertex *x* and let $T'_i(x)$ be the multi-labeled tree that is identical to $T_{i}(x)$ except that no labels are assigned to its root *x*. Let *G*(*a*, *b*) be the size of the maximum common tree of $T_1(a)$ and $T_2(b)$. We now define for those vertices $a\in V_1,~b\in V_2$, such that $M(a)=b$, the function $G':V_1 \times V_2 \rightarrow \mathbb {N}$ as the size of the maximum common tree between subtrees $T'_{1}(a)$ and $T'_{2}(b)$ (more specifically the number of common labels between $T'_{1}(a)$ and $T'_{2}(b)$—by definition excluding the labels of *a* and *b* themselves). Notice that *G*(*a*, *b*) is not necessarily equal to $G'(a, b)$, since (i) if *a* and *b* do not correspond to each other $G'(a,b)$ is undefined, and (ii) $L_a$ or $L_b$ are not necessarily empty. Rather, as will be shown below, $G(a,b) = \max \nolimits _{(x,y) \in V_1(a)\times V_2(b)}[G'(x,y) + {\text {D}}(a, b, x, y)]$. The choice of vertices *x* and *y* corresponds to the choice of vertices which are mapped to each other and has the minimal depth among all such vertices in $T_1$ and $T_2$.

The key observation of our algorithm is that the computation of $G'(a, b)$ can be reduced to finding a maximum “cost” matching for an auxiliary graph. Let $a_1, \ldots , a_n$ be the children of *a*, and $b_1, \ldots , b_m$ be the children of *b*. The structure conditions on mapping provide the guarantee that all vertices which are leaves of downward paths from *a* without internal crucial vertices, lie in distinct subtrees. Using the Observation 5 this implies that each such vertex lies in distinct subtrees with roots $a_1, \ldots , a_n$ and $b_1, \ldots , b_m$. We know inductively that $G(a_i, b_j) = \max \nolimits _{c \in V(T_1(a_i)), d \in V(T_2(b_j))}(G'(c, d)+{\text {D}}(a_i,b_j,c,d))$.

**Fig. 5:**
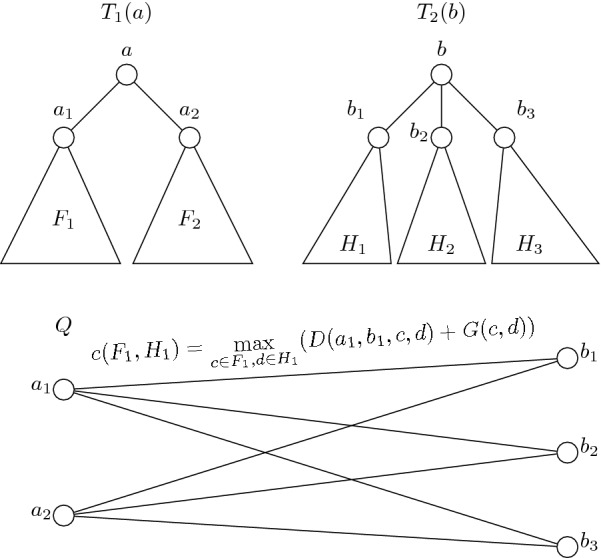
Trees $T_1(a)$, $T_2(b)$ and a graph *Q* constructed for a subproblem $G'(a, b)$ from Theorem 1

Consider now all possible bijections *N* between equal sized subsets of $\{a_1, \ldots , a_n\}$ and $\{b_1, \ldots , b_m\}$. Then $G'(a, b) = \max \nolimits _{N}\sum \nolimits _{(x, y) \in N}G(x, y)$. The problem of choosing an optimal *N* thus trivially reduces to the well known maximum weighted bipartite matching problem, which can be solved in a polynomial time [34]. For that we can construct a bipartite graph on the set of vertices $a_1, \ldots , a_n$ and $b_1, \ldots , b_m$ with the cost of an edge $(a_i, b_j)$ equal to $G(a_i, b_j)$ and return the score of an optimal assignment in this graph (with $n+m$ vertices and *nm* edges) as $G'(a, b)$. Note that if one or both of *a* or *b* are leaves then $G'(a,b)=0$. See Fig. 5 as an illustration of constructing graph *Q*. We provide an example of how our algorithm works in Appendix 2.

The time to construct auxiliary graphs is bounded by $O(|V_1||V_2|I_1I_2)$. The computational bottleneck of this algorithm is however the bipartite matching procedure: for a graph with *n* vertices and *m* edges it takes $O(nm\log {n})$ time. Let $n_a$ be the number of children of any vertex *a* in $T_1$ and $n_b$ the number of children of any vertex *b* in $T_2$; then the total time of our algorithm is $O(\sum \nolimits _{a,b}(n_a + n_b)n_an_b\log (n_a + n_b))$ which is $O(|V_1||V_2|(|V_1|+|V_2|)\log (|V_1|+|V_2|))$ or $O((|V_1|\sum \nolimits _{b}{n_b^2}+|V_2|\sum \nolimits _{a}n_a^2)\log (|V_1|+|V_2|))$. The second bound is significantly better if the maximum degree of a vertex is bounded by a small value. $\square$

## Discussion and an application

### The existing measures and their limitations

There are number of measures in the literature that are being used to compare clonal trees. Two of the most widely used measures include: (1) Ancestor–Descendant Accuracy (ADA), measure which considers only mutations originating at vertices (clones) which are in ancestor–descendant relationship in the true tree and returns the fraction of pairs of such mutations for which the relationship is preserved in the inferred tree. (2) Different-Lineage Accuracy (DLA), defined analogously as ADA, where only pairs of mutations originating from different clones which are in neither ancestor–descendant nor descendant–ancestor relationship are considered. In addition to these two measures, used in [10–12, 35] and elsewhere, (3) Clustering Accuracy (CA) [10] and (4) Co-Clustering Accuracy (CCA) [35] were also introduced in order to measure the accuracy in the placement of mutations originating from the same clone in true tree. CA measures the fraction of label pairs that are both co-located in the same vertex in both trees, whereas CCA measures the proximity in the inferred tree of pairs of mutations originating from the same clone in true tree (see [10] and [35] for definitions of CA and CCA). Finally, (5) Pair-wise Marker Shortest Path “dissimilarity” (PMSPD) [13] is (symmetric) “dissimilarity” measure calculated as the sum, over all label pairs, of the absolute difference of path length between the two labels in true tree with the equivalent length calculated in the inferred tree.

**Fig. 6:**
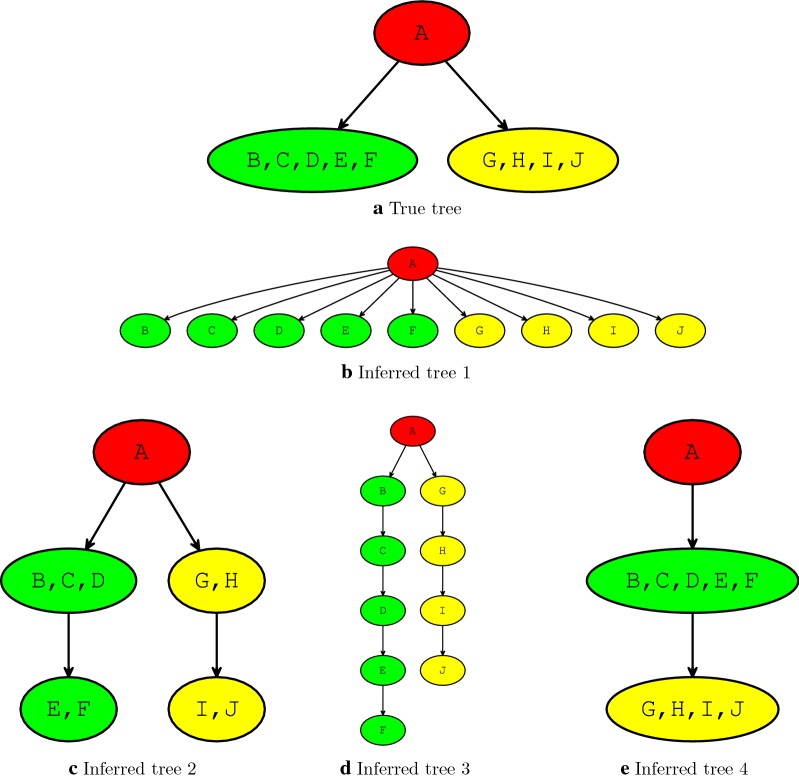
**a** True clonal tree depicting the evolution of hypothetical tumor.** b**–**e** Hypothetical trees inferred by methods for reconstructing history of tumor evolution (input data to these methods is assumed to be obtained from the hypothetical tumor mentioned in the description of ‘True tree’). These trees are used as examples which demonstrate limitations of the existing measures for calculating similarity/“dissimilarity” between true and each of the four inferred trees (details provided in "The existing measures and their limitations" section). In "Application to the synthetic examples with the 56 available ground truth" section we discuss the application of MLTD in calculating similarities between these pairs of trees

All of the above mentioned are designed to compare inferred tree against the given true tree and no single measure can capture the overall similarity/difference between two arbitrary trees. Furthermore, for each of the measures there exist cases where it returns high similarity for topologically very different true and inferred trees. We will illustrate this below by presenting several examples using trees from Fig. 6 where true tree and four trees inferred by (hypothetical) methods are shown. Each vertex in any one of these trees have one or more labels (corresponding to mutations in clonal trees) represented by $A, B, C, \ldots , J$.

**Fig. 7:**
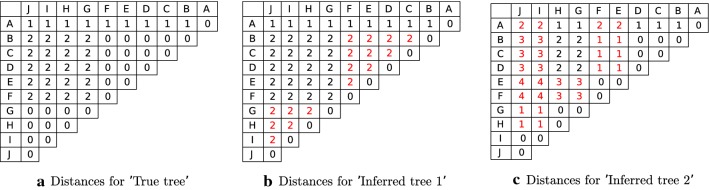
“Dissimilarities" between pairs of labels required for calculating Pair-wise Marker Shortest Path “dissimilarity” (PMSPD) for trees from Fig. 6. Entries in each matrix represent length of path between labels (note that labels are shown in the first row and the first column of each matrix). “Dissimilarity” is calculated as the sum of absolute values of differences between pairs of entries which are at the same position in both matrices. Red colored entries in labels pairwise “dissimilarity” matrix shown in** b**,** c** differ from the corresponding entries in matrix for true tree shown in **a** and therefore contribute to the overall “dissimilarity”. PMSPD assigns the same score to ‘Inferred tree 1’ and ‘Inferred tree 2’, despite the fact that ‘Inferred tree 2’ is, from the perspective of interpreting tumor evolution, much closer to ‘True tree’

For ADA measure, one needs to consider all pairs of labels in the true tree: $\{(A, B), (A, C), (A, D), (A, E), (A, F), (A, G), (A, H), (A, I), (A, J)\}$. We see that ‘Inferred tree 1’ has the maximum score despite being topologically very different from ‘True tree’. The same tree can be used as an illustration for the limitations of DLA measure where the following set of label pairs need to be considered in true tree $\{(B, G), (B, H), (B, I), (B, J), (C, G), (C, H), (C, I), (C, J), (D, G), (D, H), (D, I), (D, J), (E, G), (E, H), (E, I), (E, J), (F, G), (F, H), (F, I), (F, J)\}$. Clustering of mutations in ‘Inferred tree 4’ is in the perfect agreement with the clustering in the ‘True tree’ hence both CA and CCA measures will return maximum score for this tree, even though it is also topologically very different from ‘True tree’. Finally, the calculation of the PMSPD measure between the ‘True tree’ and ‘Inferred tree 1’, as well as ‘Inferred tree 2’, is shown in Fig. 7. This measure assigns the same score to these two inferred trees, despite the fact that ‘Inferred tree 2’ is, from the perspective of interpreting tumor evolution, much closer to ‘True tree’.

### Applications of MLTD

In order to facilitate the interpretation of results, for two arbitrary trees $T_{1}$ and $T_{2}$, in addition to the MLTD similarity measure which returns the number of mutations in common tree of $T_{1}$ and $T_{2}$ and is denoted here as $MLTD(T_{1}, T_{2})$, we also introduce $\text {MLTD-normalized}(T_{1}, T_{2})$ defined as $\frac{MLTD(T_{1}, T_{2})}{\max (a,b)}$, where *a* and *b* denote number of mutations in $T_{1}$ and $T_{2}$. MLTD-normalized can be interpreted as similarity measure which takes values from [0, 1], with higher values denoting higher similarity among trees. In the discussion of results below, all presented scores represent MLTD-normalized similarity measure, although it is obviously equivalent to MLTD (assuming that the sets of vertex labels are known for both trees, which is true in all of our comparisons).

#### Application to the synthetic examples with the available ground truth

In this section we discuss similarity between true and inferred trees shown in Fig. 6.

‘Inferred tree 1’ has relatively low score equal to 0.3 which rewards the proper placement of mutation A and correctly inferred phylogenetic relations for pairs of mutations originating from different clones, but penalizes for extensive branching which leads to the inaccurate placement to different branches of mutations originating from the same clone, as well as to significant topological differences between this and true tree. In contrast, and as expected based on our discussion from the introduction, ‘Inferred tree 2’ (which represents slightly refined version of ‘True tree’ where green and yellow clones are each split into two adjacent clones belonging to the same branch) and ‘Inferred tree 3’ (which represents fully resolved mutation tree that can be obtained from ‘True tree’) both have score 1. ‘Inferred tree 4’, having score 0.6, is rewarded for the proper placement of mutation A and large cluster of mutations appearing for the first time at green clone, but is penalized for inaccurate placement of yellow clone from where 4 out of 10 mutations originate.

#### Application to real data

**Fig. 8:**
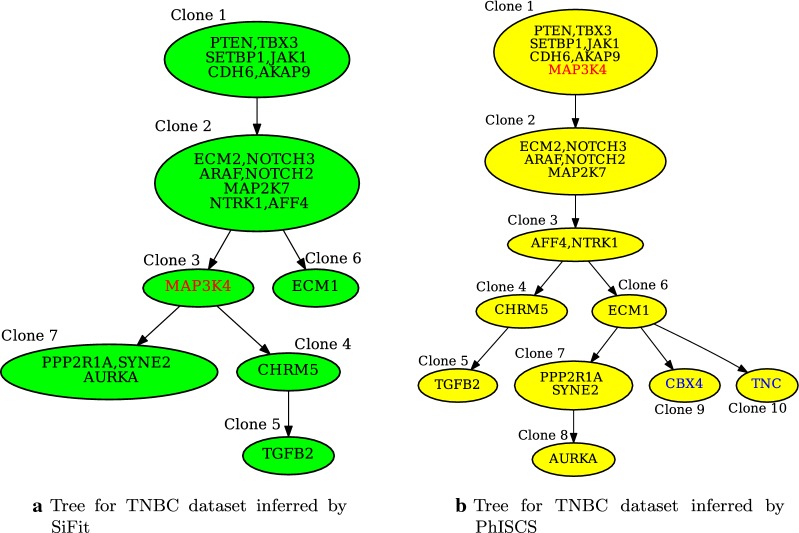
Clonal trees of tumor evolution, inferred by SiFit and PhISCS, for triple-negative breast cancer (TNBC) dataset originally published in [37] and consisting of the binary presence/absence profile of 22 mutations across 16 single cells. Names of the clones are assumed not to be included as part of the vertex label. Trees are very similar to each other in placement of the vast majority of mutations: (i) Clone 1 in the SiFit tree is almost identical (with respect to the set of mutations assigned to its label) to Clone 1 in PhISCS tree (ii) Clone 2 in SiFit tree is split into two adjacent clones, namely Clone 2 and Clone 3, in PhISCS tree. Analogous applies to Clone 7. (iii) The order of mutations in genes CHRM5 and TGFB2, as well as in most other pairs of mutations (including the pairs where both mutations are at the same vertex), is same among the trees. Notable exceptions leading to some dissimilarities between the trees include mutations in genes MAP3K4 and ECM1. In addition, mutations in genes CBX4 and TNC are absent in tree reported by SiFit. Removing these four mutations and their corresponding vertices from each tree (if present) and assigning each of the Clone 4 and Clone 7 in SiFit tree as child of Clone 2, and Clone 7 as child of Clone 3 in PhISCS tree, we obtain trees which are same up to the existence of splits of single into two adjacent clones belonging to the same lineage (see (ii) from above). MLTD-normalized score for the two trees equals 0.82, which well reflects the overall high topological similarity and concordance in ordering pairs of mutations

**Fig. 9:**
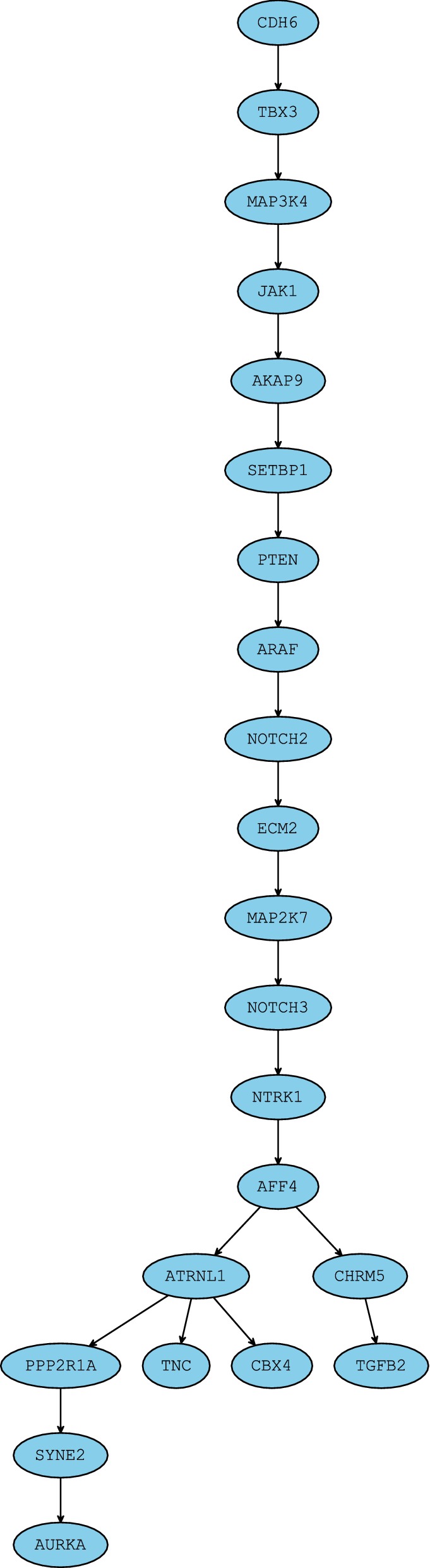
Mutation tree for TNBC dataset (see Fig. 8 for details) inferred by SCITE. This tree can be obtained from PhISCS tree by expanding vertices having more than one label, hence MLTD-normalized score between the two trees is maximum possible (i.e. equals 1). Compared with tree inferred by SiFit, SCITE tree has analogous topological similarities and differences as tree inferred by PhISCS, and MLTD-normalized score for these two trees is also equal to 0.82

**Fig. 10:**
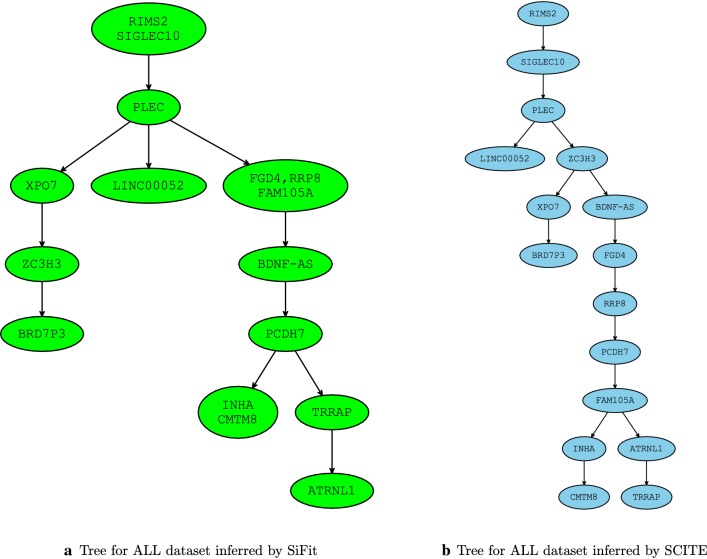
Trees inferred by SCITE and SiFit for acute lymphoblastic leukemia (ALL) patient dataset from [38] consisting of 115 single cells and 16 mutations. Unsurprisingly, due to large number of single-cells in this dataset, sequencing noise and similarities in the scoring schemes used in PhISCS and SCITE (see Appendix 1: Details of obtaining trees of tumor evolution for the real data sets) both methods report the same mutation tree so we only focus on SCITE in this discussion. The most notable difference among the two trees is in the placement and ordering of mutations in genes ZC3H3, XPO7 and BRD7P3 as well as in the ordering of mutations in genes FGD, RRP8, FAM105A, BDNF-AS and PCDH7. Furthermore, the relative order also differs for mutations in genes TRRAP and ATRNL1. However, in contrast to these important differences, the trees still share most of the major branching events in tumor evolution and have consistent ancestor–descendant order for most of the pairs of mutations. All these are reflected in MLTD-normalized score of 0.69 assigned to this pair of trees

In order to demonstrate the application of measure developed in this work in real settings where true tree is usually not available, we analyzed two datasets obtained by sequencing real samples of triple-negative breast cancer (TNBC) and acute lymphoblastic leukemia (ALL). For each sample, we inferred trees of tumor evolution by the use of SCITE [5], SiFit [3] and PhISCS [36]. We provide more details about these methods and parameters used in running them, as well as details of obtaining real data, in Appendix 1. Inferred trees and very detailed discussion of the calculated MLTD-normalized scores for pairs of inferred trees are shown in Figs. 8, 9 (for the TNBC sample) and Fig. 10 (for the ALL sample). We show that MLTD-normalized score recognizes high similarity in the placement of vast majority of mutations between two trees (as demonstrated for trees inferred by PhISCS and SiFit for TNBC sample where score equals 0.82), but also penalizes for topological differences and different sorting of mutations along linear chains (as demonstrated for trees inferred by SCITE and SiFit for ALL sample where the score equals 0.69).

## Figures and Tables

**Fig. 11:**
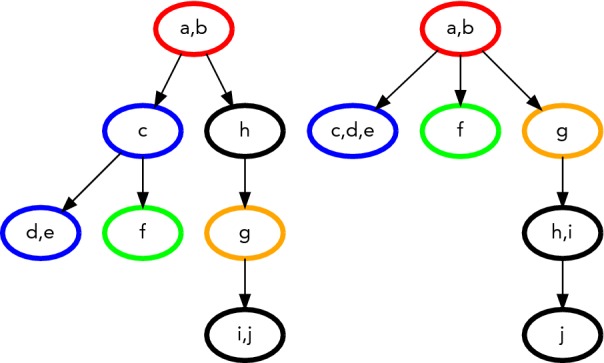
Two multi-labeled trees, $T_{1}$ (on the left) and $T_{2}$ (on the right) to be compared

## Data Availability

We have implemented our algorithm to compute MLTD and successfully applied it to a variety of data sets. The source code of the implementation can be found at: https://github.com/khaled-rahman/MLTED.

## Appendix 1: Details of obtaining trees of tumor evolution for the real data sets

### Summary of methods used for inferring trees of tumor evolution

In this work, we inferred trees of tumor evolution by the use of SCITE [5], SiFit [3] and PhISCS.^3^ Each of the methods takes as the input single-cell sequencing (SCS) data matrix and estimated noise rates of SCS experiment. The underlying scoring used in PhISCS is analogous to that in SCITE and the major difference among the two methods is in the type of tree returned in the output. While SCITE searches for the maximum likelihood mutation tree, PhISCS reports the maximum likelihood clonal tree. Due to the equivalence in tree scoring, assuming that both methods find the optimal solution, clonal tree reported by PhISCS is expected to represent compressed version of mutation tree reported by SCITE (i.e. we expect that tree reported by SCITE belongs to the set of mutation trees which can be obtained from clonal tree reported by PhISCS). Similarly as PhISCS, SiFit also returns clonal tree of tumor evolution but uses different tree search methodology and does not necessarily yield the same output as PhISCS nor SCITE (as demonstrated in [3]).

### Details of obtaining input data and running SCITE, SiFit and PhISCS

We obtained binary SCS data mutation matrix for TNBC patient sample from [39] and for ALL patient sample from [38]. For each sample, false positive and false negative rates of sequencing experiment were estimated in the original studies [37, 38] and provided as the input to the methods used in the analysis. In order to obtain better convergence, we run MCMC based methods SiFit and SCITE for very large number of iterations. For SiFit, we set number of iterations to 5,000,000. For SCITE we set number of repetitions of the MCMC to 3 and chain length of each MCMC repetition to 1,000,000. PhISCS is combinatorial optimization based method which provided guarantee of the optimality for each solution.

## Appendix 2: Demonstration of algorithm with an example

In this section, we will illustrate how the maximum common tree of trees from Fig. 11 is found using our algorithm. For the convenience of notation, since each label appears at exactly one vertex, we will use a string of concatenated labels as a unique identifier of a vertex. For example, vertex having labels *c*, *d*, *e* (blue vertex in the second tree) will be denoted as *cde*.

The first part of our algorithm consists of computing 4-dimensional table *D* (see Lemma 3 for a definition). As computation of *D* is a straightforward application of the dynamic programming, here we will skip details of this step and proceed directly to demonstrating how table $G'$ is computed. As defined earlier, for any pair of vertices $x\in V_{1},~y\in V_{2}$, such that $M(x)=y$, $G'(x,y)$ represents the size of the maximum common tree between subtrees rooted at *x* and *y*, namely $T'_{1}(x)$ and $T'_{2}(y)$—excluding the root labels. Since our algorithm for computing $G'$ is recursive, we present how $G'$ is computed in several steps, starting from the leaves of the two trees and then propagating towards the roots.

The first step for computing $G'$ is shown in Table 1. In this step, all entries $G'(u,v)$ of $G'$, where either *u* is a leaf in $T_{1}$ or *v* is a leaf in $T_{2}$, are computed (as 0 by definition).

**Table 1:** The table $G'$ with entries computed for the cases when vertex from the first tree is a leaf (such vertices are *de*, *f* and *ij*) or vertex from the second tree is a leaf (such vertices are *cde*, *f*, and *j*)

$G'$	{ab}	{cde}	{f}	{g}	{hi}	{j}
{ab}	–	0	0	–	–	0
{c}	–	0	0	–	–	0
{h}	–	0	0	–	–	0
{de}	0	0	0	0	0	0
{f}	0	0	0	0	0	0
{g}	–	0	0	–	–	0
{ij}	0	0	0	0	0	0

Rows and columns of the table respectively correspond to the vertices from the first and the second tree

Next, we compute $G'$ for a pair of vertices each with a single child (specifically, vertices $\{h\}$, $\{g\}$ in tree $T_{1}$, and $\{g\}$, $\{hi\}$ in $T_{2}$) easily—since the optimal matching contains only one edge (see Table 2).

**Table 2:** The table $G'$ updated for entries corresponding to vertices from $T_{1}$ and $T_{2}$ each with a single child

$G'$	{ab}	{cde}	{f}	{g}	{hi}	{j}
{ab}	–	0	0	–	–	0
{c}	–	0	0	–	–	0
{h}	–	0	0	2	1	0
{de}	0	0	0	0	0	0
{f}	0	0	0	0	0	0
{g}	–	0	0	2	1	0
{ij}	0	0	0	0	0	0

Every other entry in the table correspond to a nontrivial subproblem which involves the computation of a maximum matching. First we consider the cases where one of the vertices has a single child. In this case computing the cost of maximum cost matching is relatively simple as one needs to compare the single child of the root of one tree to every child of the root on the other tree (see Table 3).

**Table 3:** The table $G'(u, v)$ with entries (*u*, *v*) updated for the cases when either *u* or *v* (but not both) has only one child

$G'$	{ab}	{cde}	{f}	{g}	{hi}	{j}
{ab}	–	0	0	3	1	0
{c}	–	0	0	0	0	0
{h}	3	0	0	2	1	0
{de}	0	0	0	0	0	0
{f}	0	0	0	0	0	0
{g}	2	0	0	2	1	0
{ij}	0	0	0	0	0	0

At this point in the example, the only entries that remain to be computed are $G'(ab, ab)$ and $G'(c, ab)$. For these entries we need to solve non-trivial instances of maximum cost matching. Recall that the construction of an auxiliary graph requires information about the values of some entries in the table *G*, where entry *G*(*u*, *v*) is defined as the size of maximal common tree between subtrees $T_1(u)$ and $T_2(v)$. Also recall that $G(a, b) = \max \nolimits _{c \in V(T_{1}(a)), d \in V(T_{2}(b))}(G'(c, d)+{\text {D}}(a,b,c,d))$ and for computing entries of *G* we just need to pick the maximum value among a few values which were computed before. Finally recall the connection between *G* and costs in the auxiliary graph for computing $G'(u, v)$: the auxiliary graph is a complete bipartite graph with the children of *u* on one side and children of *v* on the other side such that the cost of any given edge (*a*, *b*) is *G*(*a*, *b*).

The entries *G*(*u*, *v*) are given in Tables 4, 5. [We skip the details on how these entries are computed since they follow from the values $G'(u, v)$.] Let’s first focus on computing $G'(c, ab)$. For this case, the relevant auxiliary (bipartite) graph edge costs *G*(*u*, *v*) are provided in Table 4. We can now compute the value of $G'(c, ab)$: as the maximum cost matching between the children of *c* in $T_{1}$ and the children of *ab* in $T_{2}$ is between (1) *de* in $T_{1}$ and *cde* in $T_{2}$ as well as (2) *f* in $T_{1}$ and *f* in $T_{2}$, the total cost of the matching is $2+1=3$—implying that $G'(c, ab)$ is also equal to 3.

**Table 4:** The costs of relevant edges in the auxiliary graph for computing the value of $G'(c, ab)$: note that the cost for the edge (*u*, *v*) is exactly value of *G*(*u*, *v*), the size of the maximum common subtree between $T_{1}(u)$ and $T_{2}(v)$

*G*	{cde}	{f}	{g}
{de}	2	0	0
{f}	0	1	0

For computing $G'(c, ab)$ we only need the values *G* between each child of vertex *c* in $T_{1}$ (i.e. *de* and *f*) and that of vertex *ab* in $T_{2}$ (i.e. *cde*, *f*, *g*)

**Table 5:** The costs of relevant edges in the auxiliary graph for computing the value of $G'(ab, ab)$: we only provide those values for edges between each child of vertex *ab* in $T_{1}$ (i.e. *c* and *h*) that of vertex *ab* in $T_{2}$ (i.e. *cde*, *f*, and *g*)

*G*	{cde}	{f}	{g}
{c}	3	1	0
{h}	0	0	3

For $G'(ab, ab)$, the relevant edge costs of the auxiliary graph can be found in Table 5. Again, it is easy to see that the cost of the maximum cost matching and thus the value of $G'(ab, ab)$ is equal to 6. This completes the computation of the table $G'$ as presented in Table 6.

**Table 6:** The table $G'$ after filling all entries

$G'$	{ab}	{cde}	{f}	{g}	{hi}	{j}
{ab}	6	0	0	3	1	0
{c}	3	0	0	0	0	0
{h}	3	0	0	2	1	0
{de}	0	0	0	0	0	0
{f}	0	0	0	0	0	0
{g}	2	0	0	2	1	0
{ij}	0	0	0	0	0	0

**Table 7:** The slice of the 4-dimensional array *D*(*x*, *y*, *u*, *v*) if we fix the first two index values as $x = ab$ and $y = ab$

*D*(*ab*, *ab*)	{ab}	{cde}	{f}	{g}	{hi}	{j}
{ab}	2	2	2	2	2	2
{c}	2	3	2	2	2	2
{h}	2	2	2	2	2	2
{de}	2	5	2	2	2	2
{f}	2	3	3	2	2	2
{g}	2	2	2	3	4	4
{ij}	2	2	2	3	4	5

Here the entry in the *u*-th row and *v*-th column represents *D*(*ab*, *ab*, *u*, *v*)

There is one last step to obtain the final answer, i.e. the value of *G*(*ab*, *ab*). For that recall that $G(a, b) = \max \nolimits _{c \in V(T_{1}(a)), d \in V(T_{2}(b))}(G'(c, d)+{\text {D}}(a,b,c,d)).$ Thus if we want to compute the value *G*(*ab*, *ab*) we should use the values from the array *D* which contains answers on the corresponding instances of the set alignment problem. In the following table, we present these values (answers on instances of the set alignment problem). Combining Tables 6, 7 we compute the value of *G*(*ab*, *ab*) to be equal to $8 = 6 + 2$.

## Footnotes

1 Given two input trees, the size of their largest common tree (with the restriction that only leaf labels can be deleted) can be used as a similarity measure. This similarity measure is akin to set intersection and MLTD, as its dual, is akin to the symmetric difference between sets. As per symmetric difference between sets, MLTD does not satisfy the triangle inequality and thus does not form a “metric”.

2 Note that typically edit distance measures are based on symmetric edit operations, in a way that each operation is complemented by a reverse operation (e.g. deleting a label is the reverse of inserting the same label). In such cases, the edit distance is defined as the minimum number of operations required to transform one combinatorial object into another. Although it is possible to define our dissimilarity measure similarly (with label insertions complementing label deletions), we chose to present our dissimilarity measure by specifying deletions only for keeping the description compact.
